# Diagenesis as the main control of clayrock brittleness

**DOI:** 10.1038/s41598-026-43512-w

**Published:** 2026-03-18

**Authors:** Adrien Damon, Roger Soliva, Christopher Wibberley, Jade Dutilleul, Sylvain Grelaud, Frédéric Bourgeois

**Affiliations:** 1 Géosciences Montpellier, CNRS, Université de Montpellier, 34000 Montpellier, France; 2 TotalEnergies, CSTJF, 64000 Pau, France

**Keywords:** Clayrock, Brittleness, Diagenesis, Sealing, Storage, Energy science and technology, Engineering, Solid Earth sciences

## Abstract

Geological storage is expected to play an important role in the energy transition, in various forms spanning across CO_2_ sequestration and nuclear waste storage to that of short-term energy (H_2_ or compressed air energy storage, CAES). The efficiency of geological storage relies on the low permeability of clayrocks, whose integrity might be compromised if they exhibit brittle behavior. Therefore, identifying the geological conditions that lead to brittleness is essential to guide storage projects toward the safest options. Here, we show that diagenesis is the main control on clayrock brittleness and that it can be predicted using first order parameters accessible from exploration borehole data. We evidence a strong correlation between clayrocks unconfined compressive strength and burial-induced diagenesis, driven by compaction and cementation resulting from clay-mineral transformation. We suggest that mechanically-consolidated clayrocks may retain ductile, low-permeability behavior if they remain confined, while cementation favors their brittle behavior. The trend we present enables the estimation of brittleness and permeability behavior of clayrocks based on their burial history. We propose that this trend can help guide the selection of safe storage sites when integrated into a dedicated exploration framework.

**Supplementary Information:**

The online version contains supplementary material available at 10.1038/s41598-026-43512-w.

## Introduction

Clayrocks are the most common seals or caprocks in geological storage projects due to their low permeability and lateral extent in sedimentary basins. There are many studies dedicated to the characterization of their permeability behavior^1,2^, driven by the industrial demand of storage solutions, particularly in the context of radioactive waste management^3^ and carbon storage^4^. Aside from the fact that clayrocks feature low permeability and low porosity^2,5^, concerns have been raised regarding their brittleness^6–8^ and subsequent permeability development^9,10^, leading to a loss of containment. It is therefore essential to identify the geological conditions resulting in a brittle behavior in order to identify the safest storage options. Among the numerous studies proposing brittleness indices^11^, clay content^12,13^ and exhumation^7,8^ are generally considered the main geological factors that control rock brittleness. However, clayrock brittleness is likely affected by other geological factors such as diagenesis, porosity and effective stress^14^. Recent work on the subject suggest a control by diagenetic processes^14,15^ and that clay content alone is insufficient to determine brittleness for various clayrocks of distinct origin^16^. However, there exists no work in the literature that provides a quantitative confrontation of clayrock strength, mineralogy and burial, which are relevant to discuss diagenesis. This barrier prevents a geomechanical vision of the geological conditions resulting in clayrock brittleness, required to build a predictive framework for storage site assessment. The large amount of data acquired on clayrocks from the Oil & Gas sector, URLs (underground research laboratories) and experimental laboratories up to the present day offers an opportunity to address this problem. Using this dataset, we analyze together clayrock strength, mineralogy, porosity, burial, and exhumation in a quantitative manner, and discuss the role of diagenesis on clayrock brittleness. To do so, we use the unconfined compressive strength (UCS) as an index of mechanical behavior frequently used to define brittleness indices^1,5,7,11^.

## The lack of precision of geology-based brittleness indices

**Fig. 1:**
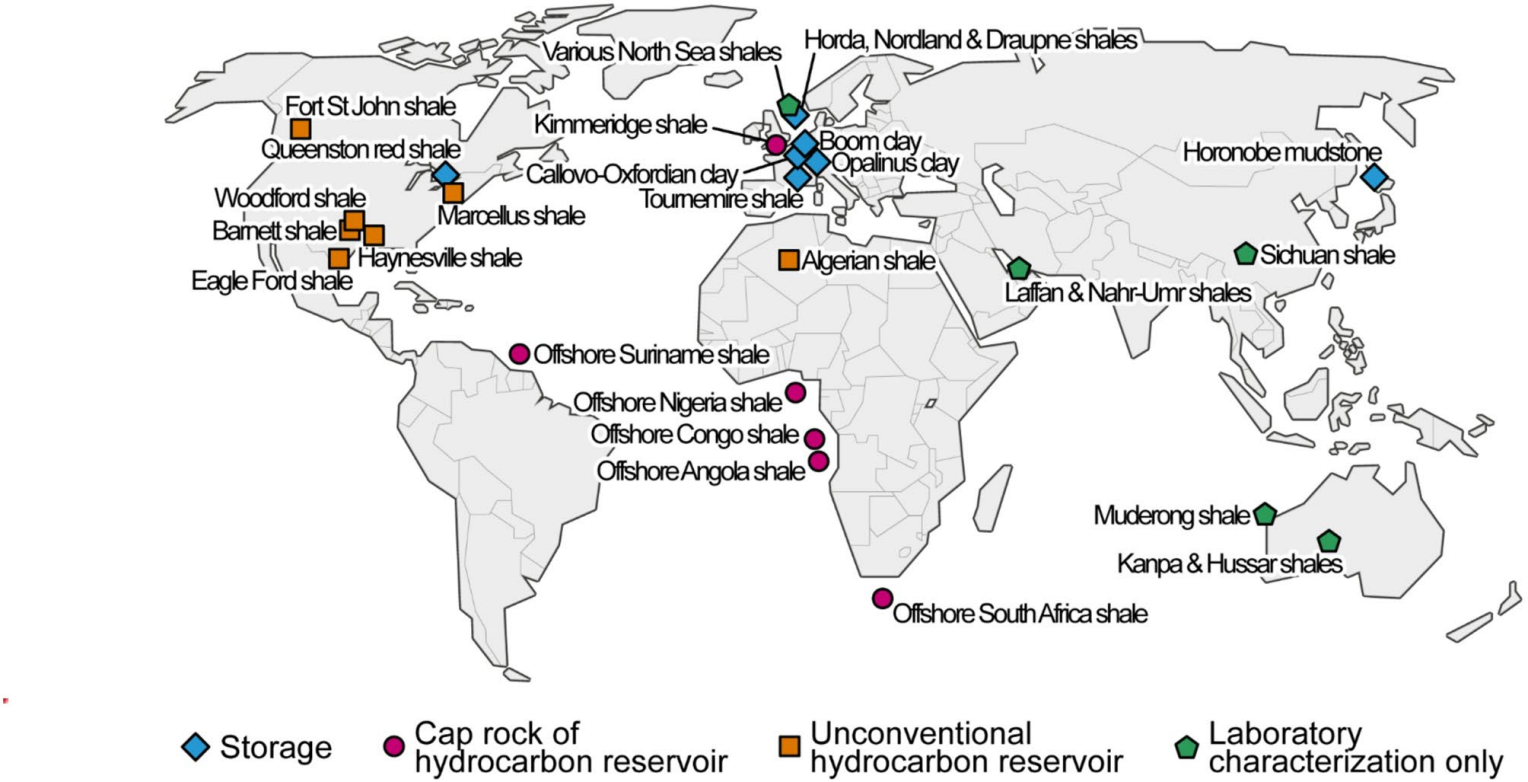
Location and use of the studied clayrocks. Map modified from https://en.wikipedia.org/wiki/File:Simple_world_map.svg using Adobe Illustrator (https://www.adobe.com/products/illustrator.html, version 30.1).

**Fig. 2:**
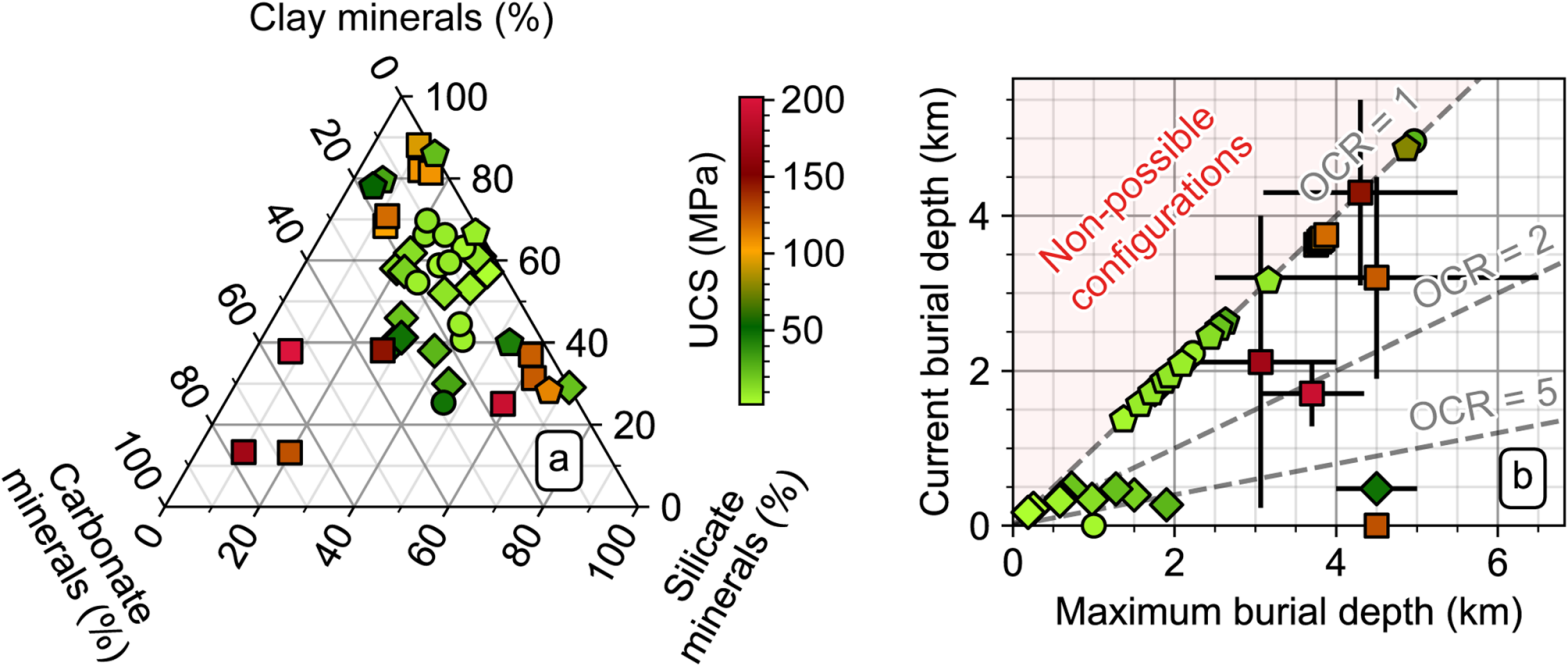
UCS of clayrocks depending (**a**) on their mineralogical composition and (**b**) on their exhumation represented by the OCR parameter (dashed grey lines), according to their maximum and current burial depths (uncertainty indicated by the black plain lines). The shape of the markers indicates their use as detailed in Fig. 1.

Indices based on clay content rely on the fact that quartz and carbonates are more brittle than clay minerals, and hence that their relative content in a clayrock should indicate brittleness^12,13^. Indices based on exhumation suggest that the decrease in confining pressure associated with exhumation may induce a change in clayrock behavior from ductile to brittle^7,8^. However, a synthesis of the currently available data of intact clayrock samples (Fig. 1, see Methods and Supplementary Material for details and references) shows no clear correlation between UCS and either the mineralogical composition or the degree of exhumation (Fig. 2). The latter is expressed in terms of the overconsolidation ratio (OCR), defined as the ratio of the maximum past vertical effective stress to the current vertical effective stress level. These two parameters therefore do not provide a definite insight into brittleness evaluation, from a mechanical point of view. Firstly, clayrocks of very similar mineralogical compositions may display UCS values spanning over two orders of magnitude (Fig. 2a). For example, among the studied clayrocks, clay-rich samples (clay minerals content between 60 and 90%) exhibit UCS values between 10 and 120 MPa. Clayrocks of lower clay content (between 20 and 40%) and similar contents in carbonate and silicate minerals exhibit UCS values between 25 and 200 MPa. Secondly (Fig. 2b), the studied clayrocks exhibit similar UCS values when their maximum burial depths are close, independent from their OCR, be they non-exhumed (OCR = 1) or strongly exhumed (OCR > 2, OCR > 5). The OCR indeed is a factor of embrittlement, but a direct correlation to this parameter appears impossible. However, the UCS of the studied clayrocks appears to increase drastically with maximum burial, from values of less than 50 MPa for maximum burial depths lower than 3 km, to values up to 100–200 MPa for maximum burial depths greater than 3 km (Fig. 2b). These observations suggest that factors other than clay content control the strength, and hence the brittleness of clayrocks, and that their strength-brittleness couple is acquired during burial.

## The role of burial diagenesis

**Fig. 3:**
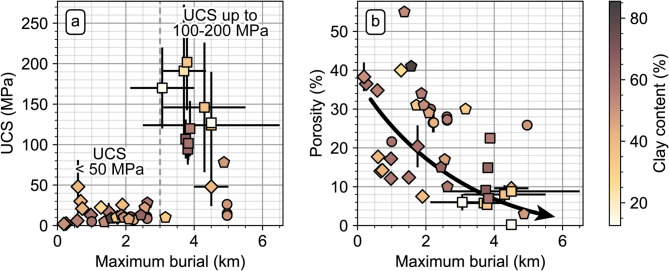
UCS (**a**) and porosity (**b**) as a function of maximum burial for the studied clayrocks, with color indicating the clay content. The shape of the markers indicates their use as detailed in Fig. 1. The black arrow in (**b**) in schematical.

We reveal a strong increase of the maximum UCS of the studied clayrocks relative to maximum burial depth, associated with a decrease in porosity (Fig. 3). Clayrocks subjected to less than 3 km of burial feature UCS values smaller than 50 MPa, while clayrocks that have been buried deeper than 3 km feature UCS values that can be more elevated and reach 100–200 MPa (Fig. 3a). Furthermore, over the range of 0–5 km of maximum burial depth, porosity roughly decreases from 30 ± 20% to less than 10% (Fig. 3b). No clear control by the clay content on the evolution of strength and porosity of the studied clayrocks is apparent. This is indicated by the lack of trends relative to their heterogeneous clay content over the studied maximum burial range over which their UCS and porosity evolve (Fig. 3).

For studied clayrocks that have been buried between 187 m and 2.63 km, the UCS increases almost linearly from 2 MPa to 20 ± 10 MPa (Fig. 3a). This increasing strength of the sediments results from their progressive compaction by grain rearrangement^17^ as indicated by their decrease in porosity^18^ (Fig. 3b). In this depth range, the porosity of the sediments allows them to behave like soils, in a ductile manner^14^, and brittle strain localization is presumably inhibited during deformation^19^. However, brittleness and dilatant fractures may be induced if compacted sediments undergo decompression^17^, particularly if they are exposed to effective stress levels lower than their UCS^1,7,20^ ($\frac{\sigma^{\prime}}{UCS}$ lower than 0.5–1, with $\sigma^{\prime}$ the effective stress). In this case, the brittleness of the compacted sediments is manifested as fissility^21^.

**Fig. 4:**
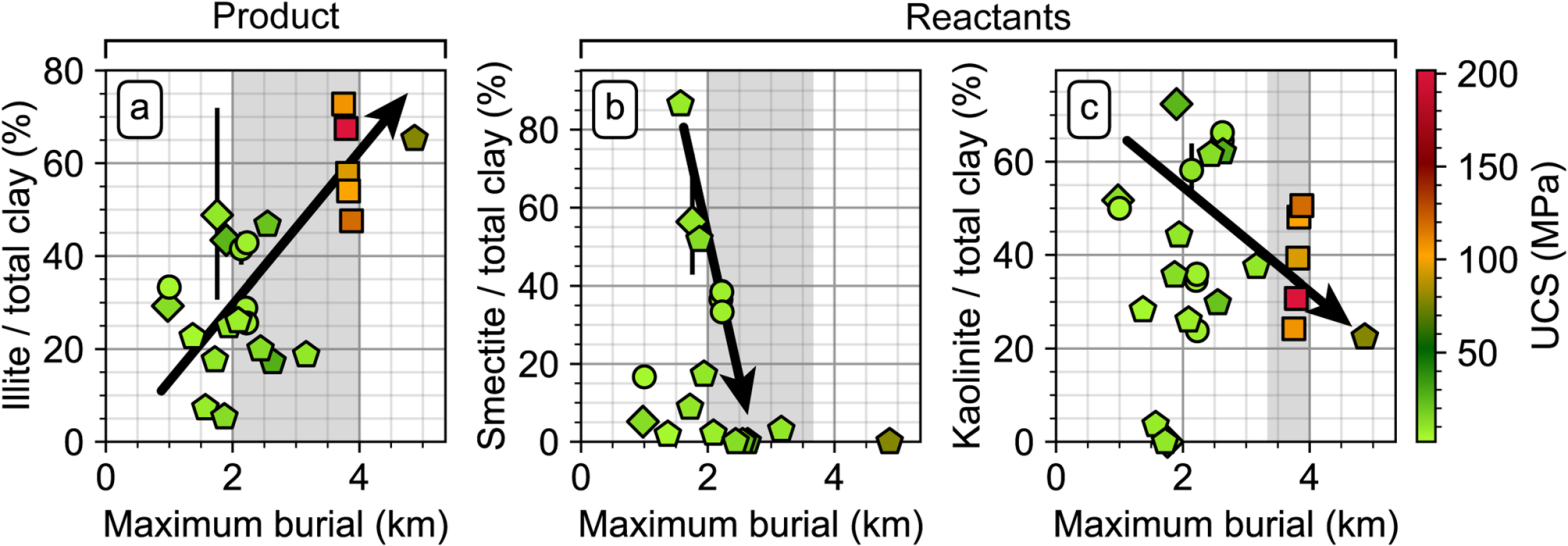
Ratios of illite (**a**), smectite (**b**) and kaolinite (**c**) relative to the total clay content of the studied clayrocks, as a function of their maximum burial, and UCS. The grey area indicates the depth interval for which the respective clay mineral forms (illite) or reacts (smectite and kaolinite), assuming a geothermal gradient of 30 °C/km. The shape of the markers indicates their use as detailed in Fig. 1. The black arrows are schematical.

For studied clayrocks that have been buried beyond 3 km, the UCS values are more heterogeneous in the range of ~ 20–200 MPa, with no particular trend relative to maximum burial. At these depths, the sediments are exposed to temperatures allowing the transformation of clay minerals into illite and quartz (between 60 and 120 °C)^22,23^, and pervasive quartz cementation occurs^24^. The increase in illite content with increasing maximum burial among the studied clayrocks, accompanied by the decrease of smectite and kaolinite content (Fig. 4), provides evidence for the onset of chemical reaction and authigenic cementation. This reaction coincides with a UCS increase from ~16 ± 12 MPa for clayrocks with ~10–50% of illite relative to total clay content (maximum burial smaller than 3 km), to ~140 ± 60 MPa for clayrocks with ~50–75% of illite relative to total clay content (maximum burial larger than 3 km) (Fig. 4a). This increase in UCS is attributed to the strengthening effect of cementation^25^. Here again, the cemented sediments may exhibit brittle behavior and dilatant fractures if their stress loading is lower than their UCS^1,7,20^. The cleavage of the sediments acquired during the compaction phase is likely enhanced by the increase in particle alignment associated with clay-minerals conversion^26^ and by the development of further anisotropy by the emplacement of authigenic quartz^27^.

## Concluding remarks on clayrock brittleness

**Fig. 5:**
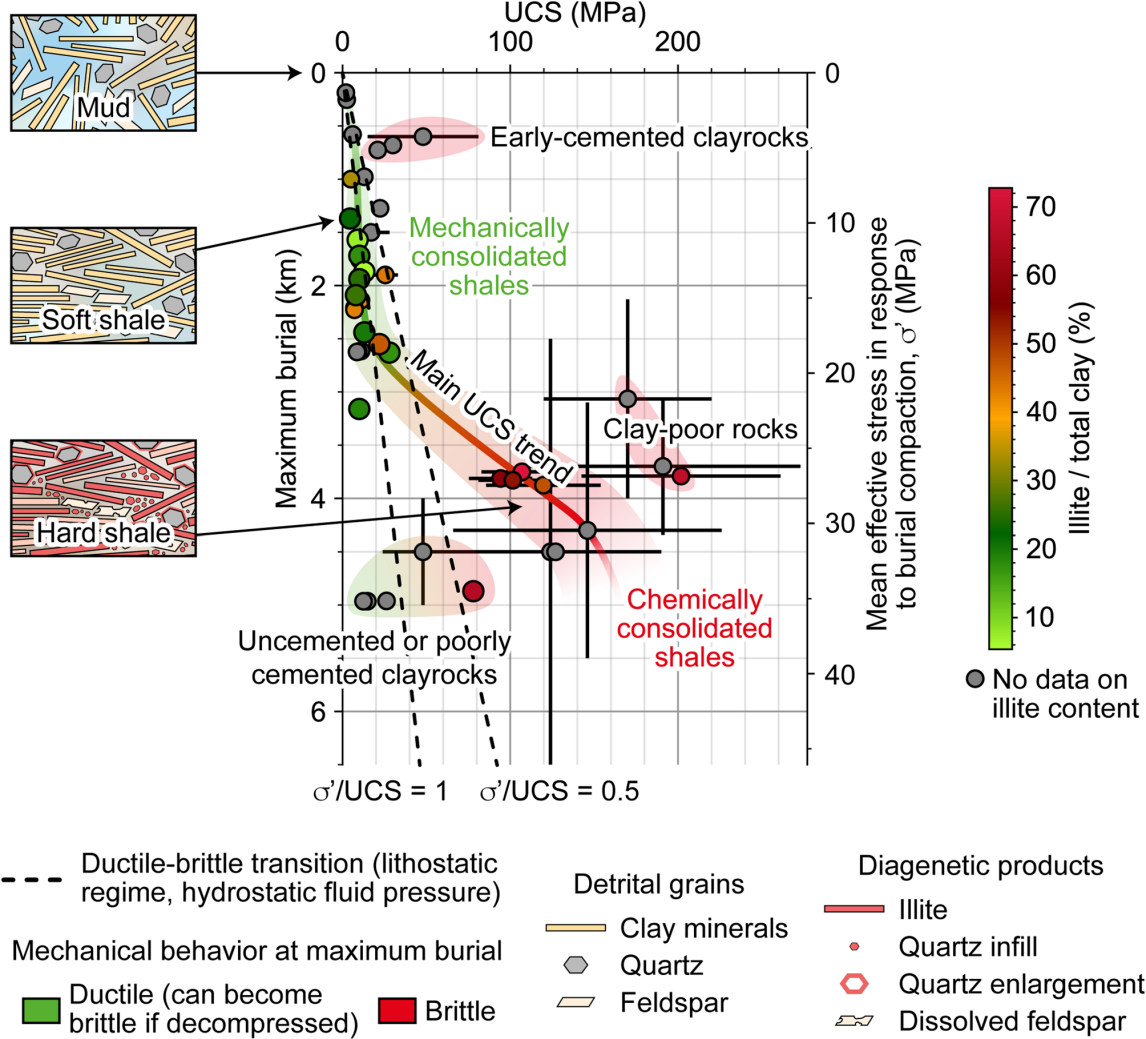
The main trend of the UCS of clayrocks relative to their maximum burial and the associated diagenetic evolution, along with the ductile-brittle transition of clayrocks in the main trend. Calculation of the mean effective stress $\sigma^{\prime}$ is presented in the Methods section.

The presented data correspond to 25 sites for which rock strength, mineralogy, porosity and burial are presented together, allowing to identify first-order trends interesting to discuss regarding geological controls on brittleness. Although there is a lack of petrographic evidences of illite conversion and mode of deformation in the literature, this dataset suggests that the main observed UCS-burial trend can be accounted by diagenetic processes (Fig. 5). Indeed, strengthening and porosity loss are generally caused by (1) grain rearrangement (rotation, bedding-alignment, sliding and bending of clay particles)^17^, (2) density increase by conversion of clays to illite^23^, and (3) cementation by authigenic quartz^24^. In the studied dataset, we propose that mechanical consolidation (point 1 stated above) occurs between 0 and 3 km of burial, whereas chemical consolidation (points 2 and 3 stated above) occurs between 3 and 4 km, resulting in significant UCS increase (Fig. 5). Depths of 3 to 4 km correspond to about 100 °C for a normal geothermal gradient, which is very consistent with the temperature at which chemical diagenesis (smectite and kaolinite conversion to illite and quartz) dominates mechanical diagenesis^22^.

Several factors may cause clayrocks to deviate from the main suggested trend (Fig. 5). Firstly, a low geothermal gradient may preserve sediments from the onset of chemical diagenesis, limiting consolidation to compaction. Related to this, a fast burial rate and / or relatively recent age of burial deeper than 3 km could prevent significant progress of these time-dependent chemical processes. Secondly, low content in reacting clay minerals may also significantly limit chemical consolidation. These are particularly expected in the dataset showing low UCS at large depths (> 4 km), where chemical consolidation would otherwise be expected. Thirdly, early cementation such as expected in chemically-reducing environments may exceptionally allow consolidation at shallow depth^28,29^. Finally, the presence of carbonates and/or silicates in very large proportions enhances rock strength^5,12,30^, but this effect is no more significative in case of advanced chemical diagenesis.

Clayrock brittleness can be discussed according to both consolidation (UCS) and effective stress ($\sigma^{\prime}$)^1,7,20^. The marked increase in strength observed around 3 km of maximum burial suggests a high probability of embrittlement. Assuming that values of $\frac{\sigma^{\prime}}{UCS}$ between 0.5 and 1 reflect the brittle-ductile transition in clayrocks^1,7,20^, chemical consolidation provides a sufficient increase in UCS to transit from conditions of ductile to brittle behavior (path to the right in Fig. 5). According to this criterion, the domain of ductile behavior expands linearly with depth, and brittleness relies mainly on chemical processes rather than mechanical compaction. Therefore, normally consolidated clayrocks (i.e., non-exhumed) that have only undergone mechanical diagenesis (maximum burial of less than 3 km) may preserve their ductile behavior at their maximum burial depth. However, in contexts of exhumation or fluid overpressure, brittleness may increase by stress reduction (upward path in Fig. 5), consistently with the OCR concept^10,11^. Also worth considering, this suggests that tectonic compression promotes clayrock ductility^31^, which is also described for sandstones^32^.

The sealing potential of clayrocks largely relies on ductility^5^. Clayrocks suitable for geological storage are therefore those that did not pass beyond the ductile-to-brittle transition. This corresponds to normally consolidated clayrocks that have only experienced mechanical diagenesis. Their permeability may only increase if decompression occurs (exhumation, increase in fluid pressure), causing them to fall into the range of stress conditions that promote brittle behavior. Consequently, case-specific studies including both UCS estimation and a dedicated stress model are required to estimate the embrittlement of clayrocks targeted for storage.

## Methods

The database analyzed in this study has been compiled from multiple studies on mudrocks, principally shales, from 28 locations around the world with various geological contexts (epicontinental to marine deposition, exhumed and non-exhumed). The studied rocks and their characteristics are presented, with references, in Supplementary Material. The compilation consists in:

- The type of rock regarding its industrial use: storage, cap rock of hydrocarbon reservoir, unconventional hydrocarbon reservoir, or laboratory characterization only;
- The UCS of the rock and its characterization method;
- The mineralogical composition of the rock;
- The porosity of the rock, along with the porosity-measurement method and the type of measured porosity;
- The burial history of the rock.

For its analysis, the dataset is plotted in ternary and classic binary diagrams, without any more complex data manipulation.

The mean effective stress presented in Fig. 5 is estimated according to the following equations:

$${\sigma}^{{\prime}}=\frac{{\sigma^{\prime}}_{v}+{\sigma^{\prime}}_{H}+{\sigma^{\prime}}_{h}}{3}$$

With ${\sigma^{\prime}}_{v}$ the effective vertical stress caused by overlying sediments:

$${\sigma^{\prime}}_{v}=({\rho}_{sed}-{\rho}_{fluid})gz$$

And ${\sigma^{\prime}}_{H}$ and ${\sigma^{\prime}}_{h}$ the effective major and minor horizontal stresses in response to vertical, uniaxial strain^33^, i.e. the vertical compaction caused by burial:

$${\sigma^{\prime}}_{H}={\sigma^{\prime}}_{h}={\sigma^{\prime}}_{v}\times\frac{\nu}{(1-\nu)}$$

With ${\rho}_{sed}$ the density of the overlying sediments at depth $z$, set to 2300 kg/m^3^ as a mean value, ${\rho}_{fluid}$ the density of the pore fluid, set to 1000 kg/m^3^ as a reference for water, $g$ the gravitational acceleration of 9.81 m/s^2^, $\nu$ the Poisson coefficient of the sediments, set to 0.25 as a mean value.

## Supplementary Information

Below is the link to the electronic supplementary material.

Supplementary Material 1

## Data Availability

The dataset used in this study is available in the Supplementary Material.

## List of symbols

${\sigma}^{{\prime}}$: Mean effective stress
OCR: Overconsolidation ratio
UCS: Unconfined compressive strength
URL: Underground research laboratory
